# Understanding and Intervening in Nutrition-Related Health Disparities

**DOI:** 10.1016/j.advnut.2024.100195

**Published:** 2024-03-21

**Authors:** Maninder Kahlon

**Affiliations:** Department of Population Health, Dell Medical School, University of Texas at Austin, Austin, TX, United States

In their article presenting a nutrition health disparities framework, Agurs-Collins et al. [1] help us systematize where to look to understand health disparities, and how to think through distinct mechanisms of action and scales of influence to mitigate those disparities. The mechanisms range from biological, behavioral, physical, sociocultural to the health care system itself. Biologically, genetics can predispose us to allergies, or the influence of physical environments can be felt in food deserts that limit access to nutritionally rich foods. These domains of influence also interact, compounding or relieving one another. Alcohol use disorder has a genetic component, can be improved by behavioral routes, such as via Alcoholics Anonymous, and can be exacerbated by built environments that promote alcohol sales.

In addition to different domains of influence, there is a range of loci at which disparities are created, from individual, to family, community, and societal levels. Disparities that arise because of individual actions and constraints happen within broader realms of influence—interpersonal interactions, the resources they have access to in the communities where they live or work, and societally, the accumulated effects of policies that inform their history.

Such a framework is an essential tool for research, program development, and policy-making. The framework helps us broaden the limited focus of any one domain or level of influence. For example, individual-level, biological interventions, such as glucagon-like peptide 1 (GLP-1) drugs suppress appetite and food intake and have been approved for weight-influenced health conditions, such as diabetes. A wider view reminds us that they have been made available within a society that associates weight with notions of self-worth. This suggests that we might consider behavioral or sociocultural interventions, to prevent abuse.

One of the most important observations within the nutrition health disparities framework is the recognition that the healthcare system itself is a domain of influence. For example, in the management of end-stage renal disease, the health care system needs to ensure that patients consume nutritional treatments that help rebalance their metabolic environment to maximize the benefits of dialysis. But a patient’s access to health insurance influences, at an individual level, the expertise and resources they can access to address these nutrition-influenced health care needs.

Some communities have health systems dedicated to supporting nutrition needs. For example, Harris Health, the county health system in Houston, Texas, has for over a decade established healthy food pantries with culturally aligned services, even dedicating resources to a farm at the community hospital site. Living in such a community gives a patient with low income an edge over someone in a community where health care players have no such strategic investment in food and nutrition. And, of course, societal policies defining what is reimbursable by health insurance are a gate to accessing nutritional support.

The inclusion of health care as a domain of influence for nutrition-related health disparities is nuanced [2]. Health care is an industry focused on delivering medical care to patients. Providing food and nutritional resources has been seen by some as an overreach. Indeed, the activities of providing better nutrition may not always fit the expertise and infrastructure of health care. Such activities may be better suited to food banks, grocery stores, community-based social service organizations, or food, agriculture, and economic policies. The nutrition health disparities framework does not quell these questions, but unequivocally puts healthcare in the mix. After all, no other system is responsible for medical outcomes. Even with a carve-out for what public health is supposed to achieve, the prevalence of diet-influenced medical conditions, such as diabetes, kidney, and cardiovascular health means the health care system must use all potential tools to solve for these. If results can be achieved via nutritional and food-related support for patients, especially those who are otherwise simply not getting better, then we must leverage this tool. Health care also wields unparalleled resources. With ∼20% of United States Gross Domestic Product (GDP) captured in the current health system, it feels odd, even irresponsible, to look to other sectors to deploy resources to solve for medical conditions that are the purview of the health care system.

The nutrition health disparities framework has one gap that future iterations will need to consider—the food system itself. If one were discussing the pharmaceutical health disparities framework, then we might consider the structure of the pharmaceutical industry, the funding and incentives of biomedical discovery, and research as potential domains of influence. The food industry remains a black box. The nutrition health disparities framework does signal some of the potential food system influences, for example pointing to policies that disincentivize the consumption of unhealthy foods, e.g., soda taxes. But one could almost imagine a complementary framework on nutrition health disparities that takes a food systems lens on biological, behavioral, physical, and sociocultural domains of influence.

Even in the health care domain, if one considers hospital cafeteria food, the mechanisms of the food system exacerbate or improve our health [3]. If you can afford it, new healthy meal delivery services abound. But in most other settings, the nutrition landscape is characterized by cheaper, high-margin food, invariably ultraprocessed.

Broadening the aperture to include the food system would also highlight an unhealthy contradiction. The system that produces what we hope is increasingly more nutritious food is also the work environment for those who face some of the greatest health disparities [4]. Farm laborers, meat factory workers, and food service staff experience a life environment, income, and working conditions that drive health disparities, many of which are nutrition related. They also have some of the lowest rates of health insurance [5]. Future work might more explicitly connect the dots between a food system that drives nutrition-related disparities for the general population, and the nutrition-related health outcomes for those that work within it.

The nutrition health disparities framework helps us identify layered influences, allowing us to consider all possible tools to create equitable health outcomes. In the 21st century, with innovation pushing the boundaries of what human beings are able to achieve, the nutrition health disparities framework reminds us to focus on ensuring the basics so that we are all fueled to maximize our contributions to the future.
